# Association between high fear-avoidance beliefs about physical activity and chronic disabling low back pain in nurses in Japan

**DOI:** 10.1186/s12891-019-2965-6

**Published:** 2019-11-28

**Authors:** Tomoko Fujii, Hiroyuki Oka, Kenichiro Takano, Fuminari Asada, Takuo Nomura, Kayo Kawamata, Hiroshi Okazaki, Sakae Tanaka, Ko Matsudaira

**Affiliations:** 1 Department of Medical Research and Management for Musculoskeletal Pain, 22nd Century Medical & Research Center, Faculty of Medicine, University of Tokyo, 7-3-1 Hongo, Bunkyo-ku, Tokyo, 113-8655 Japan; 2 Research Center for the Health Promotion and Employment Support, Kansai Rosai Hospital, Amagasaki, Hyogo Japan; 3 Research Center for the Health Promotion and Employment Support, Osaka Rosai Hospital, Osaka, Japan; 4 Department of Rehabilitation Sciences, Kansai University of Welfare Sciences, Osaka, Japan; 5 Department of Orthopaedic Surgery, Kanto Rosai Hospital, Kawasaki, Kanagawa Japan; 6 Department of Orthopaedic Surgery, Faculty of Medicine, University of Tokyo, Tokyo, Japan

**Keywords:** Fear-avoidance beliefs, Low back pain, Nurses

## Abstract

**Background:**

High prevalence of low back pain (LBP) in nurses has been reported globally. Ergonomic factors and work-related psychosocial factors have been focused on as risk factors. However, evidence on the role of fear-avoidance beliefs (FABs) concerning LBP in nurses is lacking. This study examined LBP prevalence and the association between FABs and chronic disabling LBP that interfered with work and lasted ≥ 3 months.

**Methods:**

Female nurses (*N* = 3066; mean age = 35.8 ± 10.6 years) from 12 hospitals in Japan participated. A self-reported questionnaire was used to collect information on sociodemographics, LBP, work-related factors, and psychological distress. FABs about physical activity were assessed using a subscale from the FAB Questionnaire (score range = 0–24). The participants were asked to choose one of four statements regarding their LBP in the past 4 weeks: 1) I did not have LBP, 2) I had LBP without work difficulty, 3) I had LBP with work difficulty but without requiring absence from work, and 4) I had LBP requiring absence from work. If the participant had LBP in the past 4 weeks, it was also inquired if the LBP had lasted for ≥ 3 months. Chronic disabling LBP was defined as experiencing LBP with work difficulty in the past 4 weeks which had lasted for ≥ 3 months. In the nurses who had experienced any LBP in the past 4 weeks, we examined the association between FABs and experiencing chronic disabling LBP using multiple logistic regression models adjusting for pain intensity, age, body mass index, smoking status, psychological distress, hospital department, weekly work hours, night shift work, and the12 hospitals where the participants worked.

**Results:**

Four-week and one-year LBP prevalence were 58.7 and 75.9%, respectively. High FABs (≥ 15) were associated with chronic disabling LBP (adjusted odds ratio = 1.76, 95% confidence interval [1.21–2.57], *p* = 0.003).

**Conclusions:**

LBP is common among nurses in Japan. FABs about physical activity might be a potential target for LBP management in nurses.

**Trial registration:**

UMIN-CTR UMIN000018087. Registered: June 25, 2015.

## Background

Low back pain (LBP) is a common physical symptom, which approximately 80% of people experience at some point during their lifetime [1]. LBP is the leading cause of years lived with a disability globally [2]. Especially, a high prevalence of LBP among nurses has been reported worldwide. The reported one-year prevalence of LBP is 60–70% [3–7]. In Japan, LBP is the leading occupational ailment that requires sick leave ≥ 4 days, and the number of cases is especially high in the health and hygiene industry [8], including hospitals and nursing facilities.

The etiology of LBP is multifactorial. For LBP in nurses, ergonomic factors such as patient handling and other nursing duties have been focused on primarily as risk factors [9]. Therefore, for LBP prevention, using assistive devices like lifts and sliding boards and manual handling training to reduce the physical load on nurses’ backs have been recommended, although there is a lack of strong evidence of their efficacy [10].

Psychological factors such as distress, depressive mood, and depression are risk factors for new episodes of LBP [11] as well as for chronicity and disability [12]. Taiwanese and Australian studies have reported an association between psychological symptoms or psychological distress and LBP in nurses [13, 14]. Stress management has been included as an intervention for LBP control in nurses [15].

Among the psychosocial factors associated with LBP, fear-avoidance beliefs (FABs) is a cognitive factor that predicts the outcomes of patients with LBP [12, 16, 17]. In the fear-avoidance model, pain-related fear leads to avoidance behavior, resulting in disuse, depression, and disability [16]. A study reported that FABs predicted sickness absence in female healthcare helpers and assistants who had recently graduated from school [18]. However, most studies on the association between FABs and LBP outcomes have been conducted on patients with LBP or workers on sick leave [19]. There have been few reports regarding FABs and LBP disability among nurses working in hospitals [20]. If FABs play an important role in LBP disability in hospital nurses, they could be a target for LBP control along with ergonomic factors and psychological stress in this population. We collected data about LBP and related information as a baseline assessment in a randomized controlled trial (RCT) about LBP using a large sample of nurses across Japan, and examined the association between FABs and chronicity/disability of LBP.

## Methods

### Aim, design, and setting

The aim of this cross-sectional study was to examine 1) the four-week and one-year prevalence of LBP and 2) the association between FABs and chronic disabling LBP in nurses working in hospitals across Japan.

### Participants

This study utilized baseline data from an RCT on the effects of stretching exercises on nurses’ LBP, which was conducted from July 1, 2015 to June 30, 2016 [21]. The study was registered in the University Hospital Medical Information Network Clinical Trial Registry (UMIN-CTR) (ID: UMIN000018087). The study utilized the population approach with cluster randomization. The intervention was stretching exercises, which were expected to be conducted in the workplace, to promote the exercise habits of all nurses, and to prevent LBP as well as improve LBP disability. Nurses working in 12 hospitals across Japan were invited. The inclusion criteria were all nurses who were working in the 12 hospitals and agreed to participate in the RCT. The exclusion criteria were 1) unwillingness to participate and 2) being pregnant. As this RCT utilized the population approach, nurses without LBP at baseline were not excluded. The participants were asked to individually return the completed baseline questionnaire in a sealed envelope to reduce reporting bias. In these 12 hospitals, 3439 nurses consented to participate in the study and completed the baseline survey. Men were excluded from this analysis owing to their small number (*n* = 186). Additionally, 187 respondents were excluded owing to pregnancy or missing information on sex, pregnancy, or LBP in the past 4 weeks. Thus, 3066 nurses were included in this study.

The RCT was approved by the medical/ethics review boards of the 12 hospitals and Kansai University of Welfare Sciences. All participants provided written informed consent.

### Assessment

Data were collected through a paper-based, self-administered questionnaire. Demographic variables, such as age, sex, height, body weight, smoking status, and pregnancy, were collected. Body mass index (BMI) was calculated based on self-reported body weight and height: weight (kg)/height (m)^2^. Nurses were separated into two groups: non-overweight and overweight (BMI < 25 and BMI ≥ 25, respectively) owing to the small number of nurses with a BMI ≥ 30.

Work-related data, such as those concerning hospital departments (ward, outpatient clinic, or other), work hours per week during the past month (< 40 h, 40–49 h, 50–59 h, and ≥ 60 h), years of nursing experience, night shift work, and being in a managerial position, were also collected. Categories were combined owing to low frequencies: outpatient clinics and “other” for hospital department and 50–59 and ≥ 60 for work hours.

#### LBP

Participants were asked whether they had experienced LBP in the past 4 weeks using a question written by the researchers. LBP was defined as pain localized between the costal margin and the inferior gluteal folds lasting for ≥ 1 day that may be accompanied by leg pain or numbness [22], but excluding pain related to menstruation, pregnancy, or the common cold. The definition of LBP and a diagram with a shaded area illustrating the area of pain were provided on the questionnaire. Participants were asked to choose one of the four statements regarding their LBP status in the past 4 weeks: 1) *I did not have LBP*, 2) *I had LBP without work difficulty*, 3) *I had LBP with work difficulty but without requiring absence from work*, and 4) *I had LBP requiring absence from work*. If a nurse had LBP in the past 4 weeks, we also asked whether the LBP had lasted for ≥ 3 months. In addition, the severity of LBP in the past 4 weeks was assessed using an 11-point numerical rating scale (NRS) (0 = *no pain* to 10 = *the most intense pain imaginable*). LBP experience in the past year was also inquired about with a question and responses similar to those mentioned above, because previous studies often reported a one-year prevalence of LBP.

#### Chronic disabling LBP

Nurses who answered that they had LBP with work difficulty (response 3 or 4 to the above question) in the past 4 weeks and that their LBP had lasted for ≥ 3 months were considered to be experiencing chronic disabling LBP. Any other LBP in the past 4 weeks was classified as non-chronic disabling LBP; this included LBP without work difficulty but lasted ≥3 months or LBP with work difficulty but lasted < 3 months.

#### FABs

The Fear-Avoidance Beliefs Questionnaire (FABQ), developed by Waddell and colleagues, consists of 16 self-reported items with two subscales: FABs related to work and FABs related to physical activity [23]. In this study, we used a previously developed and validated Japanese version of the FABQ [24]. Although more evidence regarding the association between the work subscale and LBP work outcomes has been accumulated, we used the four-item physical activity subscale (FABQ-PA), which assesses respondents’ FABs about physical activity. This was chosen because the intervention in the RCT was stretching exercises which were expected to promote nurses’ exercise habits. The FABQ work subscale was not included in the questionnaire in order to reduce the burden on the participants. Responses were provided on a seven-point Likert scale ranging from 0 (*completely disagree*) to 6 (*completely agree*). Thus, total scores ranged from 0 to 24, and higher scores represented higher levels of FABs. High FABs about physical activity were defined as scores ≥15 [25].

#### Psychological distress

The Kessler Psychological Distress Scale (K-6), which is a short version of the original10-item scale [26], is commonly used to assess psychological distress. It measures distress over the prior 30 days using a five-point Likert scale (0 = *none of the time* to 4 = *all of the time*). In this study, we used a previously developed and validated Japanese version of the K-6 [27].

### Statistical analyses

Initially, the four-week and one-year prevalence of LBP and other participant characteristics were examined using descriptive statistics (i.e., means and percentages). Participants were placed into three groups based on LBP status in the past 4 weeks: nurses without LBP, those who had chronic disabling LBP, and those who had LBP other than chronic disabling LBP in the previous 4 weeks (non-chronic disabling LBP). Group characteristics were compared using chi-square tests for categorical variables and Kruskal-Wallis tests for continuous variables.

The association between high FABs (FAB-PA score ≥ 15) and experiencing chronic disabling LBP in the past 4 weeks was examined using logistic regression models. Only nurses who had experienced LBP in the past 4 weeks were included in the analyses, because the FABQ asks about participants’ beliefs regarding their own LBP, and the scores should have been higher in those who had LBP at the time of assessment compared with those who did not. The dependent variable was experiencing chronic disabling LBP as opposed to having non-chronic disabling LBP, and the independent variable was the FAB-PA score (≥ 15 vs. < 15). Model 1 was a crude model. Model 2 was adjusted for NRS of LBP in the past 4 weeks. Model 3 was further adjusted for age group (20–29, 30–39, 40–49, and ≥ 50), BMI (< 25 and ≥ 25), smoking status (non-smoker, former smoker, and current smoker), hospital department (ward and outpatient clinic/other), weekly work hours in the past month (< 40 h, 40–49 h, and ≥ 50 h), night shift work (yes or no), K-6 score (< 10, 10–14, and ≥ 15), and the 12 hospitals where the nurses worked. Odds ratios (ORs) and their 95% confidence intervals (CIs) were estimated. The number of years working as a nurse was strongly correlated with age (r > 0.80); thus, it was not included in the model. Managerial position was also not included in the final model owing to the low frequency and because the change in results was negligible after adjusting for it.

Characteristics of nurses with LBP who had missing covariable values and were excluded from logistic regression analyses were compared with those who were included using chi-square tests for categorical variables and Kruskal-Wallis tests for continuous variables.

Analyses were conducted using SAS version 9.4 (SAS Institute, Inc., Cary, NC, USA). All analyses were two-sided and *p*-values < 0.05 were considered statistically significant.

## Results

**Fig. 1:**
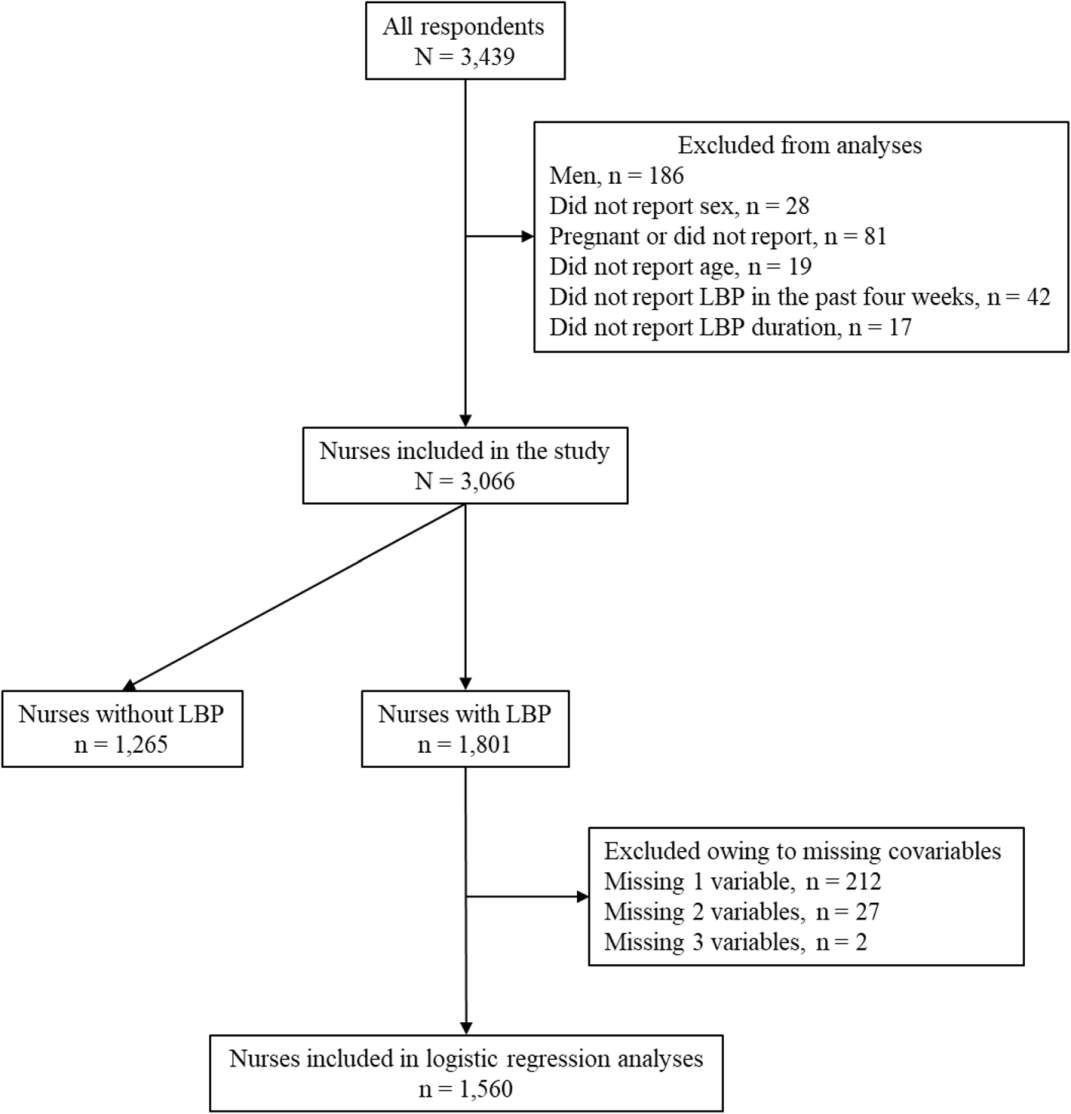
Flow of the participants

Figure 1 displays a participant flow chart. The characteristics of the 3066 nurses included in the study are shown in Table 1. The four-week and one-year prevalence of any LBP were 58.7 and 75.9%, respectively. Further, 1613 (52.7%) had non-chronic disabling and 188 (6.1%) had chronic disabling LBP at the time of assessment. For some nurses (258 out of 1265, 20.4%) the NRS value of LBP in the past 4 weeks was above 0 (minimum = 1, maximum = 7, mean = 0.5, standard deviation (SD) = 1.0), even though they answered that they did not have LBP during that time. For the majority of them (*n* = 132,) the NRS value was 1, but for one nurse, the NRS value was 7 (NRS 2: *n* = 70, NRS 3: *n* = 33, NRS 4: *n* = 15, NRS 5: *n* = 7). As LBP grades increased, so too did being overweight or obese, working in wards, working for ≥50 h/week, and working night shifts. The percentages of nurses with K6 score ≥ 10 and FABQ-PA score ≥ 15 increases with LBP grades.

**Table 1:** Participants’ characteristics (*N* = 3066)

	All	No LBP^a^	Non-chronic disabling LBP^b^	Chronic disabling LBP^c^		
	*N* = 3066	*n* = 1265	*n* = 1613	*n* = 188	Missing (n)	*p*-value
Age (years), mean (standard deviation)	35.8 (10.6)	36.0 (10.6)	35.4 (10.5)	37.5 (10.9)	0	0.021
Age (%)					0	0.108
20–29	1114 (36.3)	450 (35.6)	606 (37.6)	58 (30.9)		
30–39	837 (27.3)	335 (26.5)	454 (28.2)	48 (25.5)		
40–49	733 (23.9)	325 (25.7)	356 (22.1)	52 (27.7)		
≥ 50	382 (12.5)	155 (12.3)	197 (12.2)	30 (16.0)		
Body mass index (%)					113	< 0.001
< 25	2669 (90.4)	1148 (93.1)	1364 (88.7)	157 (86.3)		
≥ 25	284 (9.6)	85 (6.9)	174 (11.3)	25 (13.7)		
Smoking status (%)					37	0.071
None-smoker	2485 (82.0)	1047 (83.7)	1298 (81.4)	140 (76.1)		
Former	307 (10.1)	121 (9.7)	160 (10.0)	26 (14.1)		
Current	237 (7.8)	83 (6.6)	136 (8.5)	18 (9.8)		
Work experience (%)					11	0.277
< 1 year	223 (7.3)	88 (7.0)	126 (7.9)	9 (4.8)		
≥ 1 to < 2 years	190 (6.2)	83 (6.6)	96 (6.0)	11 (5.9)		
≥ 2 to < 5 years	482 (15.8)	190 (15.1)	268 (16.7)	24 (12.8)		
≥ 5 to < 10 years	522 (17.1)	230 (18.2)	260 (16.2)	32 (17.1)		
≥ 10 to < 20 years	841 (27.5)	334 (26.5)	457 (28.5)	50 (26.7)		
≥ 20 years	797 (26.1)	337 (26.7)	399 (24.8)	61 (32.6)		
Hospital department (%)					86	< 0.001
Ward	2281 (76.5)	896 (72.7)	1233 (78.8)	152 (83.1)		
Outpatient clinic/other	699 (23.5)	336 (27.3)	332 (21.2)	31 (16.9)		
Work hours (per week) (%)					70	< 0.001
< 40	455 (15.2)	216 (17.5)	219 (13.9)	20 (10.8)		
40–49	1799 (60.1)	756 (61.2)	941 (59.7)	102 (55.1)		
≥ 50	742 (24.8)	263 (21.3)	416 (26.4)	63 (34.1)		
Night shift (%)					10	< 0.001
Yes	2334 (76.4)	916 (72.5)	1263 (78.7)	155 (82.5)		
No	722 (23.6)	347 (27.5)	342 (21.3)	33 (17.6)		
Managerial position (%)					4	0.019
Yes	228 (7.5)	106 (8.4)	117 (7.3)	5 (2.7)		
No	2834 (92.6)	1157 (91.6)	1494 (92.7)	183 (97.3)		
K6 (%)					49	< 0.001
0–4	2106 (69.8)	961 (77.5)	1053 (66.1)	92 (50.3)		
5–9	619 (20.5)	192 (15.5)	373 (23.4)	54 (29.5)		
≥ 10	292 (9.7)	87 (7.0)	168 (10.5)	37 (20.2)		
FABQ-PA (%)					38	< 0.001
< 15	2143 (70.8)	1041 (83.4)	1031 (64.7)	71 (38.2)		
≥ 15	885 (29.2)	207 (16.6)	563 (35.3)	115 (61.8)		
LBP in 4 weeks (%)						NA
No LBP	1265 (41.3)	1265 (100)	0 (0)	0 (0)		
LBP without work difficulty	1580 (51.5)	0 (0)	1580 (98.0)	0 (0)		
LBP with work difficulty but without sick leave	216 (7.1)	0 (0)	30 (1.9)	186 (98.9)		
LBP with sick leave	5 (0.2)	0 (0)	3 (0.2)	2 (1.1)		
LBP NRS in 4 weeks, mean (SD)	2.2 (2.0)	0.5 (1.0)	3.1 (1.5)	5.1 (1.7)	252	< 0.001
LBP in 1 year (%)					7	< 0.001
No LBP	737 (24.1)	703 (55.7)	32 (2.0)	2 (1.1)		
LBP without work difficulty	1678 (54.9)	492 (39.0)	1179 (73.3)	7 (3.7)		
LBP with work difficulty but without sick leave	596 (19.5)	62 (4.9)	371 (23.1)	163 (86.7)		
LBP with sick leave	48 (1.6)	6 (0.5)	26 (1.6)	16 (8.5)		

*LBP* low back pain, *K6* Kessler Psychological Distress Scale, *FABQ-PA* Fear-Avoidance Beliefs Questionnaire physical activity subscale, *NRS* numerical rating scale, *NA* not applicable

^a^No LBP: Not experiencing LBP in the past 4 weeks

^b^Non-chronic LBP: any LBP in the past 4 weeks other than chronic disabling LBP

^c^Chronic disabling LBP: Experiencing LBP in the past 4 weeks that interfered with work and had lasted for ≥ 3 months

The results of the logistic regression analysis are shown in Table 2. High FABQ-PA score (≥ 15) was significantly associated with experiencing chronic disabling LBP (as opposed to having non-chronic disabling LBP) in the nurses who had any LBP during the past 4 weeks in the crude model. In Model 2, after adjusting for pain severity (NRS), the association was attenuated, but still significant. In the final multiple model further adjusting for age, BMI, smoking status, hospital departments, work hours, night shift work, K6 score, and the 12 hospitals where the nurses worked, high FABQ-PA score was still significantly associated with chronic disabling LBP (OR = 1.76 [1.21, 2.57], *p* = 0.003). All intermediate models and ORs with 95% CIs have been depicted in Additional file 1: Table S1.

**Table 2:** Association between chronic disabling LBP and fear-avoidance beliefs in nurses with LBP in 4 weeks

	Model 1		Model 2		Model 3		
	OR [95% CI]	*p*-value	OR [95% CI]	*p*-value	OR [95% CI]	*p*-value	Type 3 *p*-value
FABQ-PA ≥ 15 vs. < 15	3.19 [2.28, 4.46]	< 0.001	1.95 [1.35, 2.82]	< 0.001	1.76 [1.21, 2.57]	0.003	0.003
LBP NRS per 1 point			1.91 [1.72, 2.12]	< 0.001	1.88 [1.69, 2.10]	< 0.001	< 0.001
Age							0.122
20–29					1		
30–39					1.09 [0.66, 1.80]	0.748	
40–49					1.72 [1.03, 2.89]	0.039	
≥ 50					1.67 [0.89, 3.12]	0.110	
Overweight, yes vs. no					0.92 [0.53, 1.61]	0.774	0.774
Smoking status							0.223
None-smoker					1		
Former					1.56 [0.92, 2.66]	0.101	
Current					1.30 [0.68, 2.46]	0.428	
Clinic or other vs. ward					0.61 [0.34, 1.10]	0.101	0.101
Work hours (per week)							0.946
< 40					1.06 [0.57, 1.95]	0.854	
40–49					1		
≥ 50					1.07 [0.71, 1.61]	0.755	
Night shift, yes vs. no					1.20 [0.67, 2.13]	0.548	0.548
K6							0.267
0–4					1		
5–9					1.19 [0.77, 1.83]	0.436	
≥ 10					1.53 [0.91, 2.58]	0.109	

Chronic disabling LBP: Experiencing LBP in the past 4 weeks that interfered with work and had lasted for ≥ 3 months

Model 3: All variables and the 12 hospitals were mutually adjusted

*OR* odds ratio, *CI* confidence interval, *FABQ-PA* Fear-Avoidance Beliefs Questionnaire physical activity subscale, *LBP* low back pain, *NRS* numerical rating scale, *K6* Kessler Psychological Distress Scale

The characteristics of nurses with LBP who were excluded from logistic regression analyses owing to missing information were compared with the characteristics of those who were included. Although the nurses with LBP who were excluded from logistic regression analyses were younger (mean age = 33.4 ± 9.8 vs. 36.0 ± 10.6 years, respectively; *p* = 0.001) and tended to have higher NRS for LBP (mean NRS = 3.5 ± 1.7 vs. 3.2 ± 1.6, respectively; *p* = 0.05) compared with those who were included in the logistic models, there were no significant differences in the percentage of chronic disabling LBP, FABQ-PA scores, K6 scores, or other covariables.

In the sensitivity analysis, nurses who answered that they did not have LBP in the past 4 weeks but gave an LBP NRS value above 0 were re-classified as the “non-chronic disabling LBP” group. The estimate of the association between FABs and chronic disabling LBP did not essentially change (Additional file 2: Table S2). Similarly, when nurses who answered that they did not have LBP in the past 4 weeks but also responded that their current LBP had lasted for ≥ 3 months (*n* = 120, 9.5%) were re-classified as the non-chronic disabling LBP group, the results did not essentially change (Additional file 3: Table S3).

## Discussion

This study found that LBP was common in nurses based on a relatively large sample of female nurses working in 12 hospitals across Japan. The results also suggest that FABs might play an important role in the chronicity and disability of LBP in this group of women.

Our results are comparable to the reported one-month LBP prevalence of 54.7% among nurses working in a national university hospital in the western prefecture of Japan [28]; however, a more recent study reported a one-month LBP prevalence of 30% among nurses in a university hospital in Tokyo [29]. Smith and colleagues reported a one-year prevalence of 59%, ranging from 50 to 71% depending on the hospital department; specifically, they examined 305 nurses working in a rural Japanese university hospital [3]. In sum, the prevalence of LBP in our study was somewhat higher compared with these previous studies. We inquired about participants’ LBP status using the definition along with a diagram which was recommended as a standardized definition by Dionne et al. [22]. Despite variations in study populations, and LBP definitions, our results showed that LBP is still as common in nurses working in hospitals in Japan as in other countries [4–7].

Nonetheless, sick leave because of LBP was not common. Consistent with our results, a previous study showed that the rate of sick leave owing to musculoskeletal pain was 3% in Japanese nurses and that such sick leave was less common in Japanese workers compared with workers from the UK [29]. Another study reported that health-related costs associated with presenteeism (reduced performance while at work) were much higher than medical/pharmaceutical expenses or productivity loss associated with sick leave (absenteeism), and that LBP was the third leading cause of presenteeism following neck pain/stiff shoulders and insufficient sleep in Japanese pharmaceutical workers [30]. Although only 6% had chronic disabling LBP at the time of assessment, 21% of our participants answered that they had LBP that interfered with work sometime in the past year. Presenteeism among these nurses should not be ignored, because the shortage of nurses and their severe working conditions are significant problems in Japan. Efforts should be made to prevent and alleviate nurses’ LBP.

In nurses with LBP, those with high FABs about physical activity were about 1.8 times more likely to have chronic disabling LBP, even after adjusting for pain severity, psychological distress, work hours, night shift work, and other variables, which suggests that FABs about physical activity might be critically related to nurses’ LBP disability. Previously, focus was placed on physical risk factors for the occurrence of LBP, like heavy lifting or carrying, bending, awkward posture, and moving patients [9, 31]. In addition, the importance of psychosocial factors in LBP chronicity and disability has been recognized [12]. Work-related psychosocial factors, including high job demands, low job control, effort-reward imbalance, and low social support, were associated with LBP in nurses and nursing aides [32]. FABs, especially about work, have also been reported to predict LBP outcomes such as return to work [19]. Jensen and colleagues reported that high FABs about work were associated with sick leave days a year later and were an effect modifier between LBP severity and sick leave days in healthcare assistants and helpers who recently graduated in Denmark [18]. In their cross-sectional study of 203 hospital employees in Japan, after adjusting for various work-related psychosocial factors, Yoshimoto and colleagues reported that high FABs about physical activity were associated with having LBP that interfered with work [33]. Further, high FAB-PA scores were associated with higher LBP disability, which was assessed using the Roland-Morris Disability Questionnaire (RDQ) in Chinese and Australian nurses in a cross-sectional study by Tan et al. [20]. A systematic review also showed that FABs about work predict work-related outcomes in subacute LBP. Our results were consistent with those studies. Although there is a lack of evidence regarding whether FABs about physical activity predict work-related LBP outcomes, this and previous studies suggest that FABs about physical activity are associated with work disability among nurses, as are other psychosocial factors. Nurses are required to complete physical tasks such as handling patients, which might be a reason why FABs about physical activity were associated with work-related outcomes in our study.

For LBP control in nurses, interventions addressing ergonomic and psychosocial factors have been conducted [10, 15]. However, a Cochrane review reported no evidence on the preventative effect of manual handling training or assistive device provisions [10]. A systematic review by Van Hoof and colleagues concluded that there was no strong evidence for the efficacy of varied interventions, including manual handling training, stress management, stretching exercises, and “back school” for LBP prevention and treatment [15]. Another systematic review by Roffey and colleagues did not find evidence of a causal association between assisting patients and LBP [34], which might partially explain the scant evidence regarding the efficacy of manual handling training for LBP prevention. The results of our study suggest that FABs about physical activity might be another target for LBP control. In their pilot study, Monnin and colleagues reported that FABQ physical scores and work scores decreased significantly and remained at a six-month follow-up in healthcare workers who completed a 10-h educational program using the Back Book over 2 days, as compared with a control program [35]. Although their study did not report LBP outcomes, the intervention addressing FABs could be beneficial for the management of LBP in nurses.

One of the strengths of this study was its large sample size. Participants were nurses working in 12 hospitals across Japan. Thus, the results likely reflect the true prevalence of LBP in nurses in Japan. We collected relevant information including work hours, night shift work, and psychological distress, which are likely related to LBP. However, this study had a few limitations. Ergonomic factors regarding nursing tasks such as the frequency of patient lifting and use of assistive devices were not considered. We assessed LBP using one question with four possible responses, and LBP status was determined based on retrospective self-reports. Recall for LBP in the past year may not be accurate [36]. In addition, the nurses who expressed interest and participated in the RCT regarding LBP intervention may have overstated their LBP disability or FABs about LBP, which could also lead to bias away from the null. We were not able to exclude specific LBP, such as LBP with red flags. However, the prevalence of serious pathologies was reported to be less than 1% in patients with LBP in primary care settings [37]. In addition, the participants in our study were hospital nurses who were working at the time of assessment. Thus, not excluding LBP with red flags would not have had a large impact on our results. We did not use disability questionnaires with continuous scores, such as the RDQ or Oswestry Disability Index, to reduce the burden on the busy participants, which is also a limitation. Further, because of the cross-sectional design, the causal relationship between FABs and LBP remains unknown. Higher FABs could be a consequence of chronic disabling LBP. Future prospective studies are warranted to examine the causal relationship between FABs about physical activity and work disability among nurses with LBP. In addition, non-negligible numbers of participants were excluded from logistic regression analyses owing to missing covariable values, which might have had some influence on the results. However, most characteristics of excluded participants did not significantly differ from those who were included in analyses. Finally, as the participants were nurses working in hospitals in Japan, the results may not be generalizable to nurses in other countries, although Tan et al. found that the associations between FABs and LBP disability were similar between China and Australia [20].

## Conclusions

The prevalence of LBP remains high among Japanese female nurses. A small number of nurses had chronic disabling LBP that interfered with their work. In the nurses who had any type of LBP, high FABs were significantly associated with experiencing chronic disabling LBP. Targeting FABs about physical activity could be beneficial for LBP management in nurses.

## Supplementary information

**Additional file 1: Table S1.** Association between chronic disabling LBP and fear-avoidance beliefs in nurses with LBP in 4 weeks.

**Additional file 2: Table S2.** Results of the first sensitivity analysis.

**Additional file 3: Table S3.** Results of the second sensitivity analysis.

## Data Availability

All datasets have ethical or legal restrictions for public deposition owing to the inclusion of sensitive information from the human participants. Anonymized data are only available upon approval by the concerned institutional medical/ethics review boards.

## Abbreviations

BMI: Body mass index
CI: Confidence interval
FABQ: Fear-Avoidance Beliefs Questionnaire
FABQ-PA: Fear-Avoidance Beliefs Questionnaire–physical activity subscale
FABs: Fear-avoidance beliefs
K-6: Kessler Psychological Distress Scale
LBP: Low back pain
NRS: Numerical rating scale
OR: Odds ratio
RCT: Randomized controlled trial
