# Supplementary material for: Association between high fear-avoidance beliefs about physical activity and chronic disabling low back pain in nurses in Japan
Source: BMC Musculoskelet Disord. 2019 Nov 28;20:572. doi: 10.1186/s12891-019-2965-6 (PMC6883590; doi:10.1186/s12891-019-2965-6)
Supplement: Supplementary file 1 — Additional file 1: Table S1. Association between chronic disabling LBP and fear-avoidance beliefs in nurses with LBP in 4 weeks. [file 12891_2019_2965_MOESM1_ESM.docx]

Table S1. Association between chronic disabling LBP and fear-avoidance beliefs in nurses with LBP in 4 weeks

|  | **Model 1** | | **Model 2** | | **Model 3** | | **Model 4** | | **Model 5** | |
| --- | --- | --- | --- | --- | --- | --- | --- | --- | --- | --- |
|  | **OR [95% CI]** | ***p*-value** | **OR [95% CI]** | ***p*-value** | **OR [95% CI]** | ***p*-value** | **OR [95% CI]** | ***p*-value** | **OR [95% CI]** | ***p*-value** |
| **FABQ-PA ≥ 15 vs. < 15** | 3.19 [2.28, 4.46] | <0.001 | 1.95 [1.35, 2.82] | <0.001 | 1.9 [1.31, 2.75] | 0.001 | 1.85 [1.28, 2.69] | 0.001 | 1.85 [1.28, 2.69] | 0.001 |
| **LBP NRS per 1 point** |  |  | 1.91 [1.72, 2.12] | <0.001 | 1.88 [1.69, 2.09] | <0.001 | 1.88 [1.69, 2.09] | <0.001 | 1.88 [1.69, 2.09] | <0.001 |
| **K6** |  |  |  |  |  |  |  |  |  |  |
| 0–4 |  |  |  |  | 1 |  | 1 |  | 1 |  |
| 5–9 |  |  |  |  | 1.16 [0.77, 1.77] | 0.474 | 1.15 [0.76, 1.75] | 0.515 | 1.15 [0.76, 1.75] | 0.514 |
| ≥ 10 |  |  |  |  | 1.41 [0.85, 2.34] | 0.178 | 1.42 [0.86, 2.36] | 0.174 | 1.42 [0.86, 2.36] | 0.174 |
| **Age** |  |  |  |  |  |  |  |  |  |  |
| 20–29 |  |  |  |  |  |  | 1 |  | 1 |  |
| 30–39 |  |  |  |  |  |  | 0.99 [0.61, 1.6] | 0.958 | 0.99 [0.61, 1.6] | 0.959 |
| 40–49 |  |  |  |  |  |  | 1.43 [0.89, 2.29] | 0.14 | 1.43 [0.89, 2.3] | 0.142 |
| ≥ 50 |  |  |  |  |  |  | 1.32 [0.75, 2.32] | 0.332 | 1.32 [0.75, 2.34] | 0.334 |
| **Overweight, yes vs. no** |  |  |  |  |  |  |  |  | 0.99 [0.58, 1.69] | 0.959 |
| **Smoking status** |  |  |  |  |  |  |  |  |  |  |
| None-smoker |  |  |  |  |  |  |  |  |  |  |
| Former |  |  |  |  |  |  |  |  |  |  |
| Current |  |  |  |  |  |  |  |  |  |  |
| **Work hours (per week)** |  |  |  |  |  |  |  |  |  |  |
| < 40 |  |  |  |  |  |  |  |  |  |  |
| 40–49 |  |  |  |  |  |  |  |  |  |  |
| ≥ 50 |  |  |  |  |  |  |  |  |  |  |
| **Night shift, yes vs. no** |  |  |  |  |  |  |  |  |  |  |
| **Clinic or other vs. ward** |  |  |  |  |  |  |  |  |  |  |
| **12 hospitals** |  |  |  |  |  |  |  |  |  |  |
| Hospital 1 |  |  |  |  |  |  |  |  |  |  |
| Hospital 2 |  |  |  |  |  |  |  |  |  |  |
| Hospital 3 |  |  |  |  |  |  |  |  |  |  |
| Hospital 4 |  |  |  |  |  |  |  |  |  |  |
| Hospital 5 |  |  |  |  |  |  |  |  |  |  |
| Hospital 6 |  |  |  |  |  |  |  |  |  |  |
| Hospital 7 |  |  |  |  |  |  |  |  |  |  |
| Hospital 8 |  |  |  |  |  |  |  |  |  |  |
| Hospital 9 |  |  |  |  |  |  |  |  |  |  |
| Hospital 10 |  |  |  |  |  |  |  |  |  |  |
| Hospital 11 |  |  |  |  |  |  |  |  |  |  |
| Hospital 12 |  |  |  |  |  |  |  |  |  |  |

Chronic disabling LBP: Experiencing LBP in the past four weeks and that LBP had lasted for ≥ 3 months

OR, odds ratio; CI, confidence interval; FABQ-PA, Fear-Avoidance Beliefs Questionnaire physical activity subscale; LBP, low back pain; NRS, numerical rating scale; K6, Kessler Psychological Distress Scale.

Supplementary Table 1 continued

|  | **Model 6** | | **Model 7** |  | **Model 8** | | **Model 9** | | **Model 10*** | |
| --- | --- | --- | --- | --- | --- | --- | --- | --- | --- | --- |
|  | **OR [95% CI]** | ***p*-value** | **OR [95% CI]** | ***p*-value** | **OR [95% CI]** | ***p*-value** | **OR [95% CI]** | ***p*-value** | **OR [95% CI]** | ***p*-value** |
| **FABQ-PA ≥ 15 vs. < 15** | 1.83 [1.26, 2.67] | 0.002 | 1.83 [1.26, 2.67] | 0.002 | 1.8 [1.24, 2.62] | 0.002 | 1.78 [1.22, 2.6] | 0.003 | 1.76 [1.21, 2.57] | 0.003 |
| **LBP–NRS per 1 point** | 1.88 [1.69, 2.1] | <0.001 | 1.88 [1.68, 2.09] | <0.001 | 1.88 [1.69, 2.1] | <0.001 | 1.89 [1.69, 2.11] | <0.001 | 1.88 [1.69, 2.1] | <0.001 |
| **K6** |  |  |  |  |  |  |  |  |  |  |
| 0–4 | 1 |  | 1 |  | 1 |  | 1 |  | 1 |  |
| 5–9 | 1.15 [0.76, 1.75] | 0.512 | 1.14 [0.75, 1.74] | 0.532 | 1.16 [0.76, 1.77] | 0.49 | 1.15 [0.75, 1.75] | 0.526 | 1.19 [0.77, 1.83] | 0.436 |
| ≥ 10 | 1.45 [0.87, 2.4] | 0.155 | 1.44 [0.86, 2.39] | 0.163 | 1.43 [0.86, 2.38] | 0.174 | 1.41 [0.85, 2.36] | 0.185 | 1.53 [0.91, 2.58] | 0.109 |
| **Age** |  |  |  |  |  |  |  |  |  |  |
| 20–29 | 1 |  | 1 |  | 1 |  | 1 |  | 1 |  |
| 30–39 | 0.95 [0.58, 1.54] | 0.826 | 0.95 [0.59, 1.55] | 0.845 | 1 [0.62, 1.64] | 0.993 | 1.03 [0.63, 1.68] | 0.908 | 1.09 [0.66, 1.8] | 0.748 |
| 40–49 | 1.36 [0.84, 2.2] | 0.206 | 1.37 [0.84, 2.21] | 0.204 | 1.48 [0.91, 2.41] | 0.119 | 1.58 [0.97, 2.6] | 0.069 | 1.72 [1.03, 2.89] | 0.039 |
| ≥ 50 | 1.26 [0.71, 2.22] | 0.436 | 1.26 [0.71, 2.24] | 0.429 | 1.41 [0.78, 2.54] | 0.254 | 1.47 [0.81, 2.66] | 0.201 | 1.67 [0.89, 3.12] | 0.11 |
| **Overweight, yes vs. no** | 0.99 [0.58, 1.69] | 0.965 | 0.98 [0.57, 1.68] | 0.939 | 0.97 [0.56, 1.67] | 0.908 | 0.98 [0.57, 1.69] | 0.946 | 0.92 [0.53, 1.61] | 0.774 |
| **Smoking status** |  |  |  |  |  |  |  |  |  |  |
| None-smoker | 1 |  | 1 |  | 1 |  | 1 |  | 1 |  |
| Former | 1.56 [0.93, 2.62] | 0.089 | 1.56 [0.93, 2.61] | 0.091 | 1.57 [0.94, 2.64] | 0.086 | 1.57 [0.93, 2.63] | 0.09 | 1.56 [0.92, 2.66] | 0.101 |
| Current | 1.13 [0.61, 2.11] | 0.701 | 1.12 [0.6, 2.1] | 0.713 | 1.1 [0.59, 2.07] | 0.756 | 1.13 [0.6, 2.11] | 0.712 | 1.3 [0.68, 2.46] | 0.428 |
| **Work hours (per week)** |  |  |  |  |  |  |  |  |  |  |
| < 40 |  |  | 0.92 [0.51, 1.67] | 0.777 | 1 [0.55, 1.83] | 0.999 | 1.02 [0.56, 1.88] | 0.94 | 1.06 [0.57, 1.95] | 0.854 |
| 40–49 |  |  | 1 |  | 1 |  | 1 |  | 1 |  |
| ≥ 50 |  |  | 1.09 [0.73, 1.62] | 0.678 | 1.08 [0.73, 1.62] | 0.692 | 1.06 [0.71, 1.58] | 0.795 | 1.07 [0.71, 1.61] | 0.755 |
| **Night shift, yes vs. no** |  |  |  |  | 1.51 [0.93, 2.48] | 0.099 | 1.17 [0.66, 2.06] | 0.589 | 1.2 [0.67, 2.13] | 0.548 |
| **Clinic or other vs. ward** |  |  |  |  |  |  | 0.6 [0.33, 1.07] | 0.085 | 0.61 [0.34, 1.1] | 0.101 |
| **12 hospitals** |  |  |  |  |  |  |  |  |  |  |
| Hospital 1 |  |  |  |  |  |  |  |  | 1 |  |
| Hospital 2 |  |  |  |  |  |  |  |  | 1.05 [0.36, 3.05] | 0.934 |
| Hospital 3 |  |  |  |  |  |  |  |  | 1.49 [0.69, 3.22] | 0.306 |
| Hospital 4 |  |  |  |  |  |  |  |  | 1.06 [0.45, 2.52] | 0.896 |
| Hospital 5 |  |  |  |  |  |  |  |  | 1.57 [0.67, 3.67] | 0.301 |
| Hospital 6 |  |  |  |  |  |  |  |  | 0.52 [0.16, 1.7] | 0.276 |
| Hospital 7 |  |  |  |  |  |  |  |  | 1.33 [0.65, 2.7] | 0.434 |
| Hospital 8 |  |  |  |  |  |  |  |  | 0.8 [0.37, 1.75] | 0.581 |
| Hospital 9 |  |  |  |  |  |  |  |  | 1.33 [0.57, 3.14] | 0.511 |
| Hospital 10 |  |  |  |  |  |  |  |  | 0.91 [0.33, 2.47] | 0.849 |
| Hospital 11 |  |  |  |  |  |  |  |  | 0.94 [0.42, 2.1] | 0.884 |
| Hospital 12 |  |  |  |  |  |  |  |  | 1.1 [0.55, 2.22] | 0.789 |

Chronic disabling LBP: Experiencing LBP in the past four weeks and that LBP had lasted for ≥ 3 months

OR, odds ratio; CI, confidence interval; FABQ-PA, Fear-Avoidance Beliefs Questionnaire physical activity subscale; LBP, low back pain; NRS, numerical rating scale; K6, Kessler Psychological Distress Scale.

Model 10*=Model 3 in the main text
