# Supplementary material for: Association between high fear-avoidance beliefs about physical activity and chronic disabling low back pain in nurses in Japan
Source: BMC Musculoskelet Disord. 2019 Nov 28;20:572. doi: 10.1186/s12891-019-2965-6 (PMC6883590; doi:10.1186/s12891-019-2965-6)
Supplement: Supplementary file 2 — Additional file 2: Table S2. Results of the first sensitivity analysis. [file 12891_2019_2965_MOESM2_ESM.docx]

Table S2. Results of the first sensitivity analysis

|  | **Non-chronic disabling LBP^a^ (n = 1631)** | | **Chronic disabling LBP^b^ (n = 164)** | |  |  |
| --- | --- | --- | --- | --- | --- | --- |
|  | **n** | **(%)** | **n** | **(%)** | **OR [95% CI]^c^** | ***p*-value** |
| FABQ-PA |  |  |  |  |  |  |
| < 15 | 1103 | (67.6) | 61 | (37.2) | 1 |  |
| ≥ 15 | 528 | (32.4) | 103 | (62.8) | 1.79 [1.22, 2.61] | 0.003 |
| LBP NRS, mean (SD) | 2.9 | (1.5) | 5.0 | (1.7) | 1.94 [1.74, 2.16] | <.0001 |
| Age |  |  |  |  |  |  |
| 20–29 | 604 | (37.0) | 47 | (28.7) | 1 |  |
| 30–39 | 450 | (27.6) | 42 | (25.6) | 1.12 [0.67, 1.85] | 0.668 |
| 40–49 | 370 | (22.7) | 47 | (28.7) | 1.68 [1.00, 2.82] | 0.048 |
| ≥ 50 | 207 | (12.7) | 28 | (17.1) | 1.66 [0.89, 3.11] | 0.111 |
| BMI |  |  |  |  |  |  |
| < 25 | 1456 | (89.3) | 141 | (86.0) | 1 |  |
| ≥ 25 | 175 | (10.7) | 23 | (14.0) | 0.96 [0.55, 1.68] | 0.88 |
| Smoking status |  |  |  |  |  |  |
| Non-smoker | 1331 | (81.6) | 121 | (73.8) | 1 |  |
| Former | 174 | (10.7) | 26 | (15.9) | 1.57 [0.92, 2.67] | 0.097 |
| Current | 126 | (7.7) | 17 | (10.4) | 1.32 [0.70, 2.51] | 0.396 |
| Hospital department |  |  |  |  |  |  |
| Ward | 350 | (21.5) | 25 | (15.2) | 1 |  |
| Outpatient clinic/other | 1281 | (78.5) | 139 | (84.8) | 0.61 [0.34, 1.11] | 0.109 |
| Work hours (per week) |  |  |  |  |  |  |
| < 40 | 239 | (14.7) | 17 | (10.4) | 1.06 [0.58, 1.95] | 0.847 |
| 40–49 | 978 | (60.0) | 90 | (54.9) | 1 |  |
| ≥ 50 | 414 | (25.4) | 57 | (34.8) | 1.09 [0.73, 1.64] | 0.673 |
| Night shift |  |  |  |  |  |  |
| Yes | 1268 | (77.7) | 135 | (82.3) | 1.25 [0.70, 2.24] | 0.449 |
| No | 363 | (22.3) | 29 | (17.7) | 1 |  |
| K6 |  |  |  |  |  |  |
| 0–4 | 1108 | (67.9) | 80 | (48.8) | 1 |  |
| 5–9 | 357 | (21.9) | 49 | (29.9) | 1.22 [0.80, 1.88] | 0.357 |
| ≥ 10 | 166 | (10.2) | 35 | (21.3) | 1.53 [0.91, 2.58] | 0.107 |

^a^ Nurses who answered that they did not have LBP in the past four weeks but had an LBP NRS value above 0 (n = 258) without missing covariables (235 out of 258) were re-classified as the non-chronic disabling LBP group.

^b^ Chronic disabling LBP: Experiencing LBP in the past four weeks that had lasted for ≥ 3 months.

^c^ All variables and the 12 hospitals were mutually adjusted.

OR, odds ratio; CI, confidence interval; FABQ-PA, Fear-Avoidance Beliefs Questionnaire physical activity subscale; LBP, low back pain; NRS, numerical rating scale; K6, Kessler Psychological Distress Scale.
