# Supplementary material for: Association between high fear-avoidance beliefs about physical activity and chronic disabling low back pain in nurses in Japan
Source: BMC Musculoskelet Disord. 2019 Nov 28;20:572. doi: 10.1186/s12891-019-2965-6 (PMC6883590; doi:10.1186/s12891-019-2965-6)
Supplement: Supplementary file 3 — Additional file 3: Table S3. Results of the second sensitivity analysis. [file 12891_2019_2965_MOESM3_ESM.docx]

Table S3. Results of the second sensitivity analysis

|  | **Non-chronic disabling LBP^a^ (n = 1498)** | | **Chronic disabling LBP^b^ (n = 164)** | |  |  |
| --- | --- | --- | --- | --- | --- | --- |
|  | **n** | **(%)** | **n** | **(%)** | **OR [95% CI]^c^** | ***p*-value** |
| FABQ-PA |  |  |  |  |  |  |
| < 15 | 999 | (66.7) | 61 | (37.2) | 1 |  |
| ≥ 15 | 499 | (33.3) | 103 | (62.8) | 1.79 [1.22, 2.61] | 0.003 |
| LBP NRS, mean (SD) | 2.9 | (1.5) | 5.0 | (1.7) | 1.90 [1.70, 2.12] | <.0001 |
| Age |  |  |  |  |  |  |
| 20–29 | 524 | (35.0) | 47 | (28.7) | 1 |  |
| 30–39 | 416 | (27.8) | 42 | (25.6) | 1.09 [0.66, 1.81] | 0.733 |
| 40–49 | 358 | (23.9) | 47 | (28.7) | 1.66 [0.99, 2.79] | 0.053 |
| ≥ 50 | 200 | (13.4) | 28 | (17.1) | 1.65 [0.88, 3.08] | 0.118 |
| BMI |  |  |  |  |  |  |
| < 25 | 1333 | (89.0) | 141 | (86.0) | 1 |  |
| ≥ 25 | 165 | (11.0) | 23 | (14.0) | 0.95 [0.54, 1.66] | 0.861 |
| Smoking status |  |  |  |  |  |  |
| Non-smoker | 1215 | (81.1) | 121 | (73.8) | 1 |  |
| Former | 164 | (11.0) | 26 | (15.9) | 1.57 [0.93, 2.68] | 0.094 |
| Current | 119 | (7.9) | 17 | (10.4) | 1.31 [0.69, 2.47] | 0.413 |
| Hospital department |  |  |  |  |  |  |
| Ward | 335 | (22.4) | 25 | (15.2) | 1 |  |
| Outpatient clinic/other | 1163 | (77.6) | 139 | (84.8) | 0.60 [0.33, 1.09] | 0.096 |
| Work hours (per week) |  |  |  |  |  |  |
| < 40 | 211 | (14.1) | 17 | (10.4) | 1.08 [0.59, 2.00] | 0.796 |
| 40–49 | 900 | (60.1) | 90 | (54.9) | 1 |  |
| ≥ 50 | 387 | (25.8) | 57 | (34.8) | 1.08 [0.72, 1.62] | 0.728 |
| Night shift |  |  |  |  |  |  |
| Yes | 1158 | (77.3) | 135 | (82.3) | 1.25 [0.70, 2.23] | 0.452 |
| No | 340 | (22.7) | 29 | (17.7) | 1 |  |
| K6 |  |  |  |  |  |  |
| 0–4 | 1009 | (67.4) | 80 | (48.8) | 1 |  |
| 5–9 | 335 | (22.4) | 49 | (29.9) | 1.21 [0.79, 1.86] | 0.385 |
| ≥ 10 | 154 | (10.3) | 35 | (21.3) | 1.54 [0.92, 2.60] | 0.102 |

^a^ Nurses who answered that they did not have LBP in the past four weeks but answered that their current LBP had lasted for ≥ 3 months (n = 120) without missing covariables (102 out of 120) were re-classified as the non-chronic disabling LBP group.

^b^ Chronic disabling LBP: Experiencing LBP in the past four weeks that had lasted for ≥ 3 months.

^c^ All variables and the 12 hospitals were mutually adjusted.

OR, odds ratio; CI, confidence interval; FABQ-PA, Fear-Avoidance Beliefs Questionnaire physical activity subscale; LBP, low back pain; NRS, numerical rating scale; K6, Kessler Psychological Distress Scale.
